# Development and internal validation of the SMILE-FSS: a Free Sugars Screener for Australian children aged 2 and 5 years

**DOI:** 10.1017/S1368980023002380

**Published:** 2023-12

**Authors:** Lucinda K Bell, Shalem Leemaqz, Gemma Devenish-Coleman, Loc G Do, Diep Ha, Jane A Scott, Rebecca K Golley

**Affiliations:** 1 Caring Futures Institute, College of Nursing and Health Sciences, Flinders University, Adelaide 5001, Australia; 2 South Australian Health and Medical Research Institute, Adelaide, SA, Australia; 3 School of Population Health, Curtin University, Perth, WA 6102, Australia; 4 School of Dentistry, The University of Queensland, Brisbane, QLD, 4072 Australia

**Keywords:** Early childhood, Dietary assessment, Dental screener, Free sugars, Oral health, Preschool, Short screener

## Abstract

**Objective:**

To develop and internally validate a Free Sugars Screener (FSS) for Australian children aged 2 and 5 years.

**Design:**

Using data collected from a ninety-nine-item (2-year-olds) and ninety-eight-item (5-year-olds) FFQ in the Study of Mothers’ and Infants’ Life Events affecting oral health (SMILE-FFQ), a regression-based prediction modelling approach was employed to identify a subset of items that accurately estimate total free sugars intake (FSI). The predictors were grams of free sugars (FSg) for individual items in the SMILE-FFQ and child’s age and sex. The outcome variable was total FSI per person. To internally validate the SMILE-FSS items, the estimated FSg was converted to percent energy from free sugars (%EFS) for comparison to the WHO free sugars guideline categories (< 5 %, 5–< 10 % and ≥ 10 %EFS) using cross-classification analysis.

**Setting:**

Australia.

**Participants:**

858 and 652 2- and 5-year-old children, respectively, with complete dietary (< 5 % missing) and sociodemographic data.

**Results:**

Twenty-two and twenty-six items were important in predicting FSI at 2 and 5 years, respectively. Items were similar between ages with more discretionary beverage items (e.g. sugar-sweetened beverages) at 5 years. %EFS was overestimated by 4·4 % and 2·6 %. Most children (75 % and 82 %) were categorised into the same WHO free sugars category with most (87 % and 95 %) correctly identified as having < 10 %EFS in line with the WHO recommendation.

**Conclusions:**

The SMILE-FSS has good internal validity and can be used in research and practice to estimate young Australian children’s FSI and compare to the WHO free sugars guidelines to identify those ‘at risk’.

High consumption of dietary sugars is a major public health concern, having a negative impact on oral health^(1)^ and contributing to an increased risk of chronic diseases such as obesity^(2)^. Excessive intakes of added sugars is of particular concern in children^(3)^, with approximately one-quarter of Australian children having overweight or obesity^(4)^ and one-third having caries by the age of 5 years^(5)^. Frequent exposure to added sugars in the first years of life may reinforce the innate preference that infants have for sweet foods^(6)^ with taste preferences persisting throughout life and influencing dietary patterns in adulthood^(7)^. It is therefore important to understand patterns of sugars intakes in early life to inform early prevention efforts to reduce dental caries and overweight prevalence in children^(8,9)^.

Dietary free sugars, defined as sugar added to foods and beverages by the manufacturer, cook or consumer plus those naturally present in honey, syrups, and fruit juices, are the most important risk factor for dental caries^(1,10,11)^ and can contribute to excess energy intake with little nutritional benefit^(12)^. The WHO recommends that less than 10 % of total energy intake come from free sugars, with a conditional recommendation to reduce intake to less than 5 % of energy intake for additional health benefits^(1)^. However, recent analysis of data from the Australian Study of Mothers’ and Infants’ Life Events affecting oral health (SMILE) birth cohort has shown that some children (2·4 %) as young as 1 year old were exceeding the < 10 % energy from free sugars (EFS) recommendation (23 % exceeding < 5 %EFS recommendation)^(13)^, increasing to over a third at 2 years (38 % exceeding < 10 %EFS and 71 % exceeding < 5 %EFS)^(14)^, with similar findings at 5 years (37 %exceeding < 10 %EFS and 75 % exceeding < 5 %EFS, unpublished results). Thus, there is an urgent need to address excess intakes of free sugars in early childhood.

Accurate measurement of free sugars intakes (FSI) in early childhood supports the monitoring of population intakes to inform policy, the evaluation of interventions in practice and at scale, and for screening intakes within the primary healthcare setting to identify ‘at risk’ children requiring referral and intervention^(15,16)^. However, an ongoing limitation in research and practice is the lack of consistency and precision in the assessment of dental-specific dietary factors, leading to a call within the WHO Sugars Intake Guidelines for studies with improved dietary assessment methodology^(1)^. In response to this, the SMILE-FFQ was developed^(17)^. The SMILE-FFQ is a dental-specific, semi-quantitative eighty-nine-item FFQ (generating a list of ninety-nine foods) designed to capture the leading dietary contributories to dental caries risk in toddlers aged 18–30 months^(17)^. Although the SMILE-FFQ was determined to be a valid tool for assessing FSI of Australian toddlers using parental proxy report^(17)^, the length limits its use in both the research and practice settings.

Short dietary questionnaires or screeners, typically completed in less than 15 min^(18)^, provide an alternative to longer FFQ in settings where brief tools are needed^(15,16)^. Short questionnaires can be administered quickly in a variety of formats, tailored to outcomes of interest, are associated with a low respondent burden, and allow for easy comparison of intake against guidelines to provide a quick assessment of ‘risk’^(15,16)^. Although short questionnaires have been developed and validated in Australian children for assessing various aspects of diet, predominately dietary intake and diet quality^(15,16,19)^, none specifically assess FSI. Short dental-specific questionnaires that assess FSI of young children and that allow for comparison against the WHO free sugars guidelines are therefore required.

Thus, the aim of this study was to develop and internally validate a short-form Free Sugars Screener (FSS) for Australian children aged 2 and 5years using data derived from the long-form SMILE-FFQ. The objectives were to: (1) identify a subset of SMILE-FFQ questions that accurately estimate FSI from the complete set of survey questions, (2) internally validate the items by comparing *measured* FSI with *predicted* FSI and (3) internally validate the items by classifying participants’ FSI into the WHO free sugars guidelines categories (< 5 %, 5–< 10 % and ≥ 10 %EFS)^(1)^ and comparing measured categorisation with *predicted* categorisation.

## Methods

### The Study of Mothers’ and Infants’ Life Events affecting oral health

SMILE is a birth cohort study conducted in Adelaide, South Australia. The objectives, methods and cohort profile have been described elsewhere^(20,21)^. Briefly, 2147 mothers and 2181 newborns were recruited from the three major maternity hospitals in Adelaide between July 2013 and August 2014. All new mothers with sufficient English competency and with no intention of moving out of the greater Adelaide area within the following year were invited to participate. Mothers in hospitals that service lower socio-economic areas were oversampled to compensate for anticipated higher attrition rates^(21)^. Participants were invited to complete questionnaires on dental and dietary habits at recruitment, when their child reached 3 and 6 months of age, and again at 1, 2, and 5 years of age. This included the SMILE-FFQ, administered at 2 and 5 years of age. The SMILE study was approved by the Southern Adelaide Clinical Human Research Ethics Committee (HREC/50.13, approval date: 28 Feb 2013) and the South Australian Women and Children Health Network (HREC/13/WCHN/69, approval date: 7 August 2013).

### Data collection

#### The SMILE-FFQ

Development and validation of the SMILE-FFQ has been described in detail elsewhere^(17)^. Briefly, the eighty-nine-item SMILE-FFQ was constructed to capture dietary factors associated with dental caries risk in children aged 1–3 years. This included major food and beverage sources of total and free sugars and dental-protective foods such as milk products^(17)^. Participants report the frequency (from ‘never or rarely’ to ‘3 or more times per day’) and quantity of consumption for each food item in the questionnaire. Portion size options were tailored to the food in question and described using a combination of household measures (teaspoon, tablespoon and cup) and common portion sizes (piece, tub and pouch), each with a corresponding weight (g) and/or volume (ml). Four supplementary questions within the SMILE-FFQ split the coding of six food items into sixteen items so that the eighty-nine-item FFQ generates a list of ninety-nine foods. For example, in the SMILE-FFQ, a supplementary question ‘If your child eats yoghurt, do you choose reduced fat versions?’ is used to split four yogurt line items differentiated by flavour into eight foods, subdivided by both flavour and fat type (see online supplementary material, Supplemental Table S1). The SMILE-FFQ was validated against repeat 24 h recalls in an external cohort of toddlers aged 18–30 months, performing similarly at ranking individuals’ total and FSI, with a tendency to underestimate intakes in participants with lower reported 24 h recall values and overestimate intakes in those with higher reported values^(17)^, highlighting its suitability for observational studies wanting to use ranked total and FSI.

Minor adjustments were made to the SMILE-FFQ ahead of data collection when children were approximately 5 years of age. Checks were undertaken to ensure: (1) the item response wording was appropriate for the target age group, (2) the portion sizes listed reflected current supermarket products for older children and (3) the portion sizes listed were distributed around the median intakes of 4–8-year-old Australians^(22)^. Examples of wording changes included changing ‘infant formula’ to ‘junior formula’, removing ‘baby jars’ and ‘sippee cups’ from the portion size descriptors for some items, and removing the smallest portion size response option for some questions. No changes were made to the frequency response options. One item was added (coffee, e.g. ready-to-drink coffee beverages such as iced coffee, as coffee is a source of fluoride^(23)^) and two items removed (infant fruit purees and infant vegetable purees due to not being age-appropriate), resulting in eighty-eight items. As above, the additional four supplementary questions are used to split the coding of six food items into sixteen items, generating a list of ninety-eight foods at 5 years.

#### Sociodemographic data

Sociodemographic data were collected from mothers at recruitment via self-completed questionnaires. These included measures of maternal age, country of birth and educational attainment, in addition to household income, number of children at home and whether their child lived in a one- or two-parent household (Table 1). Mothers’ pre-pregnancy BMI (kg/m^2^) was calculated from self-reported height and weight and classified into weight status categories (healthy weight < 25 kg/m^2^, overweight 25–29·9 kg/m^2^ and obesity ≥ 30 kg/m^2^)^(24)^. Postcode was used to derive a measure of socio-economic status using the Index of Relative Socio-Economic Advantage and Disadvantage (IRSAD)^(25)^, one of the four Socio-Economic Indexes for Areas (SEIFA) indices that ranks areas across Australia on a continuum of social disadvantage to advantage^(20)^. Child age was calculated from date of birth and date of completion of the SMILE-FFQ at both 2 and 5 years of age.

**Table 1:** Characteristics of mother–child dyads in the samples with complete dietary and sociodemographic data at 2 and 5 years^a^

	2 years (*n* 885)		5 years (*n* 652)	
	*n*	%	*n*	%
Child characteristics				
Age at the time of questionnaire completion (years)				
Mean	2·1		5·3	
sd	0·1		0·4	
Child’s sex				
Male	469	53·0	357	54·8
Female	416	47·0	295	45·2
Child’s BMI category^b^				
Healthy weight	673	76·1	448	68·7
Overweight	71	8·0	143	21·9
Obesity	23	2·6	61	9·4
Maternal and household characteristics				
Mother’s age at child’s birth (years)				
Mean	30·5	5·0	30·8	5·1
sd				
Mother’s pre-pregnancy BMI^c,d^				
< 25 kg/m^2^	476	53·8	315	48·3
25–29·9 kg/m^2^	176	19·9	122	18·7
≥ 30 kg/m^2^	174	19·7	90	13·8
Mother’s country of birth				
Australia or New Zealand or UK	686	77·5	480	73·6
Other	191	21·6	165	25·3
Mother’s education attainment				
High school/vocational	379	42·8	254	93·0
Some university and above	506	57·2	398	61·0
Annual household income, $AUD				
< 40 000	97	11·0	65	10·0
40 001–80 000	267	30·2	208	31·9
80 001–120 000	269	30·4	216	33·1
> 120 001	245	27·7	156	23·9
Index of Relative Socio-economic Advantage and Disadvantage				
Deciles 1–2 (most disadvantaged)	203	22·9	146	22·4
Deciles 3–4	231	26·1	161	24·7
Deciles 5–6	151	17·1	120	18·4
Deciles 7–8	220	24·9	166	25·5
Deciles 9–10 (most advantaged)	73	8·2	54	8·3
Number of children at home				
1	413	46·7	317	48·6
2	310	35·0	232	35·6
≥ 3	137	15·5	84	12·9
Two-parent household				
Yes	834	94·2	616	94·5

a Data are presented as *n* (%) unless otherwise stated.

b Age and sex-specific BMI z-scores were calculated for children and classified into weight status categories (healthy weight ≥ –2 and ≤ +2 sd, overweight > +2 and ≤ +3 sd, and obesity > +3) using the WHO reference^(26)^.

c Weight status categories equivalent to BMI (kg/m^2^); healthy weight < 25 kg/m^2^, overweight 25–29·9 kg/m^2^ and obesity ≥ 30 kg/m^2^.

d Missing data *n* 118 at 2 years.

#### Child anthropometric data

Child weight and height data were collected at the 2- and 5-year physical examinations using standardised methodology and equipment, including calibrated electronic scales and medical stadiometers^(27)^. Age and sex-specific BMI z-scores were calculated for children and classified into weight status categories (healthy weight ≥ –2 and ≤ +2 sd, overweight > +2 and ≤ +3 sd, and obesity > +3) using the WHO reference^(26)^.

### Development of the SMILE Free Sugars Screener

#### Data source and variables

The SMILE Free Sugars Screener (SMILE-FSS), a short-form version of the SMILE-FFQ, was developed using data obtained from completion of the SMILE-FFQ at 2 and 5 years of the SMILE study. Data reduction techniques were employed to identify a subset of items that accurately estimate FSI in Australian children aged 2 and 5 years, derived from the long-form SMILE-FFQ. The predictor variable was measured FSI in grams calculated for each questionnaire item of the long-form SMILE-FFQ based on quantity and frequency response options. The outcome variable was total usual FSI derived from the long-form SMILE-FFQ calculated per person. A specific number of items was not identified as the target, however, to substantially reduce participant burden, fewer than 50, and ideally about 20–30 items was anticipated, consistent with other short screeners^(28–33)^.

#### Data cleaning and preparation

On return of the completed SMILE-FFQ at 2 and 5 years, data were cleaned and questionnaires with more than 5 % of line items left blank were excluded^(17)^. Microsoft Access version 15 (Microsoft Corporation, 2013) was used to link the questionnaire responses to an accompanying database developed specifically for the SMILE study which contained lookup tables of free sugars amounts (g) for the foods, quantity options and frequencies (per d) listed in the SMILE-FFQ^(17)^. This database linked FFQ responses to nutrient values for total sugars and free sugars using the AUSNUT 2011–2013 food consumption database^(34)^, from which total usual FSI in grams per d was calculated. Implausible FSI intakes were excluded, defined as values greater than 3 sd above the mean for both total and free sugars^(17)^.

### Statistical analysis

To identify a subset of FFQ items that can reasonably estimate total FSI of each child calculated from the complete set of survey questions, a regularised regression-based prediction modelling approach was employed at both 2 and 5 years. Participants with complete dietary (< 5 % missing FFQ data) and complete sociodemographic data were included (i.e. ‘complete-case analysis’). The predictors included grams of free sugars for each food in the SMILE-FFQ and child’s age and sex. Child’s weight and height were also included as predictors in the 5-year regression model to improve model fit, although not at 2 years due to the number of missing values (*n* 279, 26·7 %). A sensitivity analysis was performed with height and weight *included* at 2 years, and height and weight *excluded* at 5 years, to assess the consistency of variables selected.

Survey items (herein referred to as predictors) with near-zero variance (e.g. foods such as porridge, milk and milk alternatives, fruit (fresh, tinned and puree), cheese and custard, and drinks such as tea, coffee, cordial, vegetable juice and water), defined as less than 10 % unique values or the ratio of most common values is greater than 19, were removed prior to analyses (i.e. survey items which were rarely consumed and made limited contribution to grams of FSI (FSg) in participants) (see online supplementary material, Supplemental Table S2). A 10-fold cross-validation was performed, in which the sample was randomly split into a training (70 %) and testing (30 %) sample with the testing sample reserved for use only in the final internal validation step. In the first step of the analysis, we used regularised linear regression with Elastic Net (a combination of L1 Lasso and L2 Ridge) to select variables which are ‘important’ in predicting the outcome (i.e. total usual FSI per person). Lasso and Ridge regression works by estimating the regression parameters under a constraint which shrinks the estimates towards zero with the aim of reducing prediction variance (determined by the minimum root mean square error (RMSE)) and in the process ‘removes’ (i.e. shrinks to zero) unimportant predictors. The optimal model was chosen on the basis of minimum differences between training and testing RMSE across cross-validation runs.

To internally validate the items in the optimal model, the predicted FSg was converted to %EFS for comparison to the WHO free sugars guidelines categories (< 5 %, 5–< 10 % and ≥ 10 %EFS)^(1)^. However, as the SMILE-FFQ was not designed to capture total energy intake, Estimated Energy Requirements (EER) were used as an alternative for calculating the percentage of EER coming from FSI. The method for calculating EER at 2 years has been described previously^(14)^. That is, EER was determined from age and weight data using the Australian Nutrient Reference Values^(35)^ equation for children aged 13–35 months. At 5 years, the equation for children aged 3–8 year was used and EER determined from child age, weight and height data and applying a standard physical activity level of 1·6 (‘light activity’) due to a lack of physical activity data for the current sample. The percent of EER coming from FSI was then calculated using the following equation: %EERFreeSug = [(Sugars_g × 16·7) ÷ EER_kJ] × 100). The *measured* and *predicted* %EFS were then compared using the RMSE, and participants in the testing sample categorised into the WHO categories (< 5 %EFS, 5–< 10 %EFS and ≥ 10 %EFS)^(1)^ and compared descriptively. Sensitivity analysis was also conducted to compared the measured and predicted FSg in the optimal model using the testing sample at 2 and 5 years.

## Results

### Population studied

**Fig. 1:**
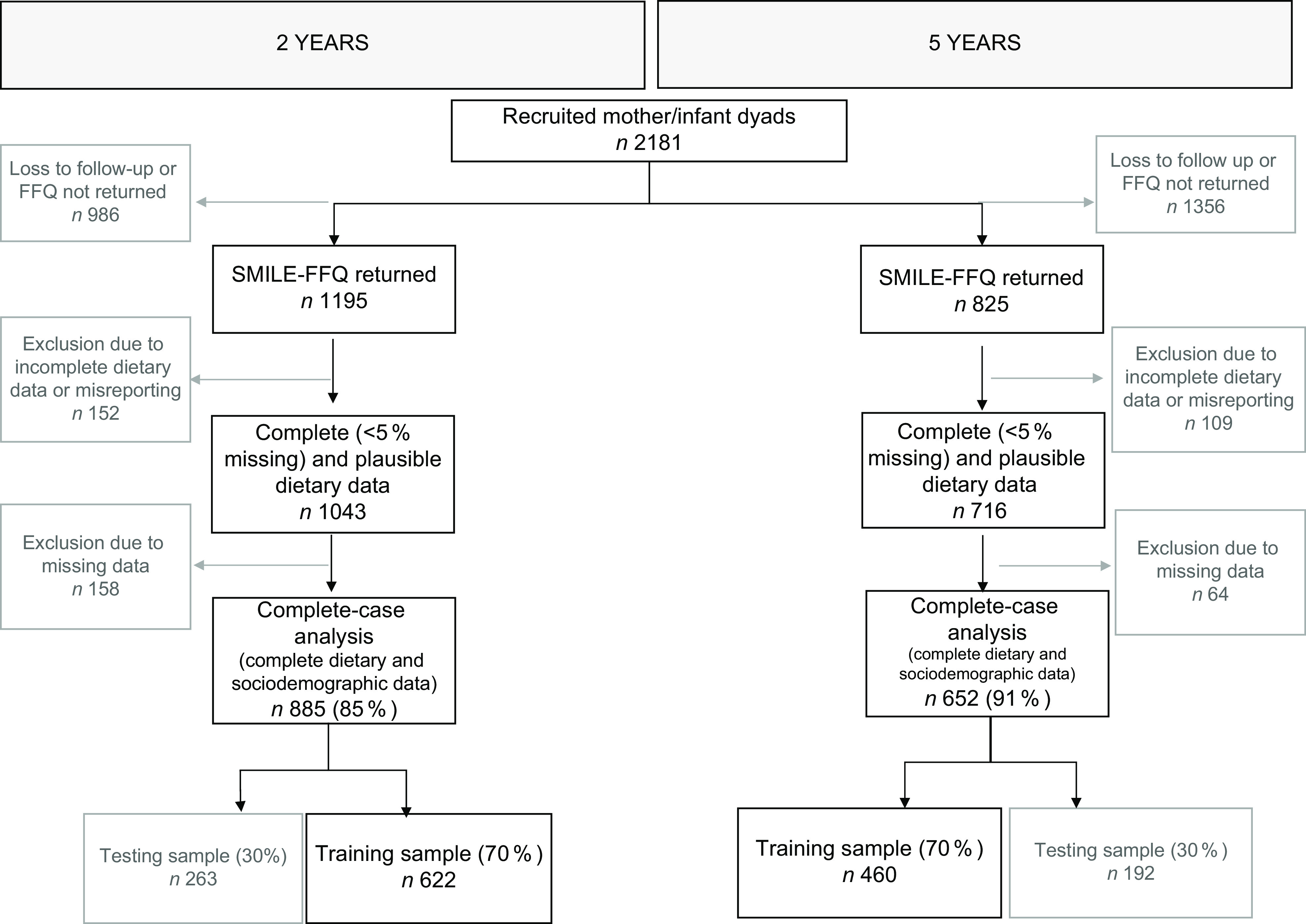
Participant flow through the study to develop a short-form version of the SMILE-FFQ. SMILE, Study of Mothers’ and Infants’ Life Events affecting oral health

Of the 2181 mothers–infant dyads recruited for the SMILE study, 1043/1195 and 716/825 SMILE-FFQs were returned complete (< 5 % missing) and plausible at 2 and 5 years, respectively (Fig. 1). Complete-case analysis was conducted on 885/1043 (2 years) and 652/716 (5 years). Sample characteristics are presented in Table 1. At the 2-year data collection wave, the mean age of children was 2·1 years (sd ± 0·1 years, range 1·9–3·2 years), with sex almost evenly split (females 47 % and males 53 %) and most being from a two-parent household (94 %). The majority of mothers (mean age 30·5 ± 5 years) were born in Australia, New Zealand or UK (78 %), with pre-pregnancy BMI less than 25 kg/m^2^ (54 %), and were university educated or higher (57 %). Similarly, nearly half of the children at the 5-year data collection wave (mean age 5·3 ± 0·4 years, range 4·7–6·5 years) were female (45 %) and approximately two-thirds from two-parent households (65 %). The majority of mothers were born in Australia, New Zealand or UK (74 %), with a pre-pregnancy BMI less than 25 kg/m^2^ (48 %), and were university educated or higher (61 %).

Supplemental Table S3 presents the sample characteristics for the complete-case samples compared to the excluded cases (i.e. those with complete dietary data but incomplete sociodemographic data). Participants excluded were largely similar to the analysed samples at 2 and 5 years; however, children from the analysed sample were slightly younger at 2 years (2·1 ± 0·1 years *v*. 2·2 ± 0·2 years, *p* = 0·03) and slightly older at 5 years (5·3 ± 0·4 *v*. 5·2 ± 0·4 years, *p* = 0·004). Mothers who provided complete data at 2 years were more likely to be born in Australia/New Zealand/UK (78 % *v*. 68 %, *p* = 0·006), while at 5 years they were less likely to be university-educated (61 % *v*. 75 %, *p* = 0·04).

### Development of the SMILE-FSS

#### SMILE-FFQ item reduction at 2 years of age

**Fig. 2:**
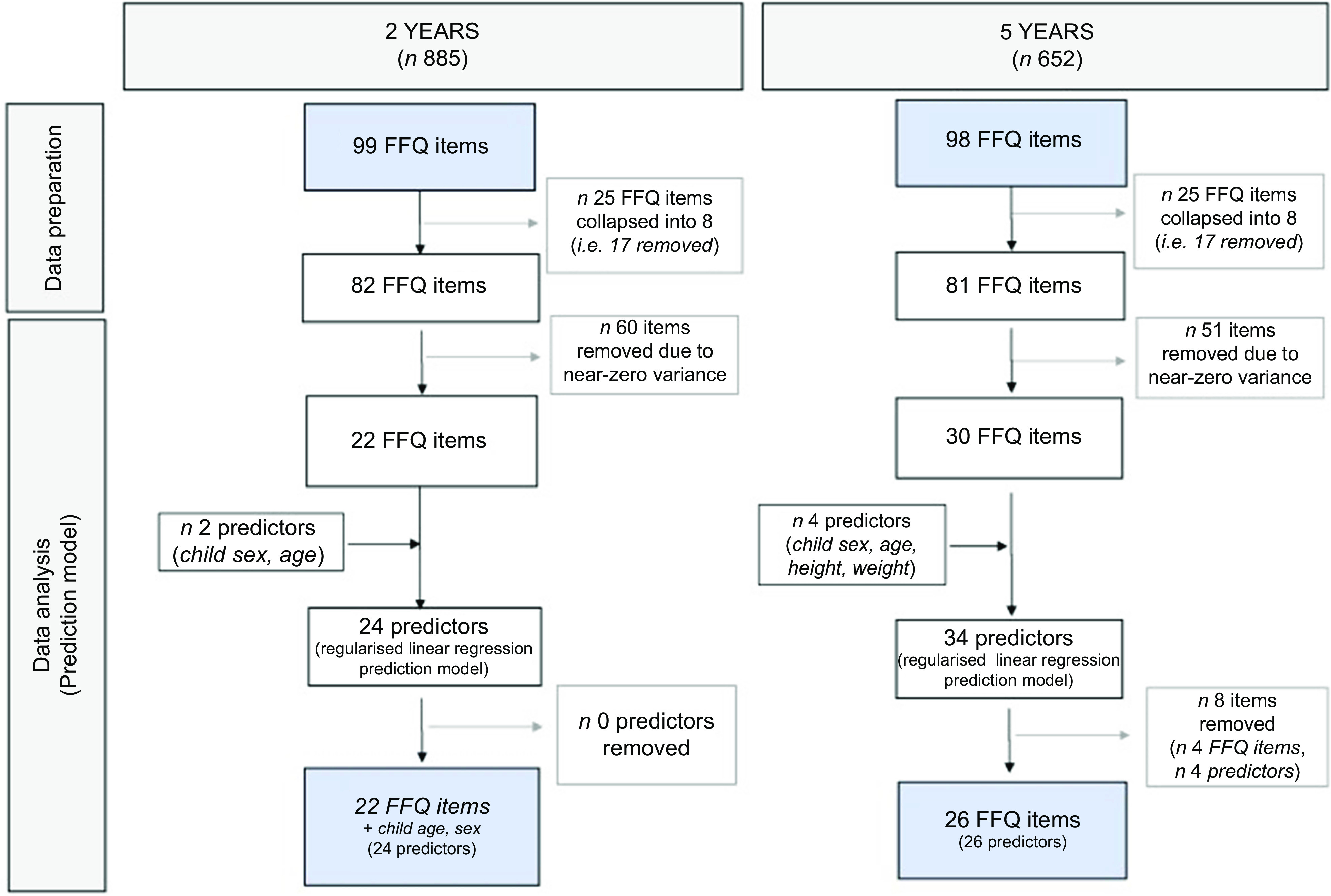
Reduction of the ninety-nine-item and ninety-eight-item SMILE-FFQ into the twenty-two-item and twenty-six-item SMILE-FSS at 2 years and 5 years, respectively. SMILE, Study of Mothers’ and Infants’ Life Events affecting oral health

Figure 2 shows the reduction of the 2-year SMILE-FFQ from ninety-nine items to twenty-two items using data from the training sample (*n* 622). First, the ninety-nine FFQ items were reduced by collapsing twenty-five FFQ items into eight (see online supplementary material, Supplemental Table S1) and subsequently removing sixty items with near-zero variance (see online supplementary material, Supplemental Table S2). A total of twenty-four predictors (twenty-two FFQ items plus child age and sex) were entered into the regularised linear regression prediction model. Table 2 shows the model coefficients after variable shrinkage across ten cross-validation runs. The RMSE across the ten runs was 2275·6 ± 28·2 (%EFS, 4·06 ± 0·26 %). The best model was R8, with an RMSE of 4·4 %EFS, meaning that on average a child’s predicted %EFS was overestimated by 4·4 %.

**Table 2:** Model coefficients after variable shrinkage across ten cross-validation runs of the regularised regression-based prediction model, using the training sample (*n* 622) at 2 years^a^

Description	R1	R2	R3	R4	R5	R6	R7	R8	R9	R10
(Intercept)	−2·452	−5·189	3·227	−1·301	0·28	−8·584	2·151	**4·445**	−6·137	−4·717
Child factors										
Child age (at 2 years)	3·695	4·535	0·527	2·71	1·957	6·055	1·705	**0·04**	5·014	4·646
Child sex	−0·773	−0·667	−0·214	0	−0·073	−0·293	−0·317	**−0·223**	−0·575	−0·67
Food frequency items										
Custard: plain or vanilla	1·331	1·314	1·398	1·391	1·123	1·334	1·242	**1·335**	1·353	1·326
Icy poles and sorbet	2·037	1·753	1·752	1·528	1·518	1·397	1·678	**1·565**	1·776	1·748
Nut paste	0	5·973	4·183	3·517	5·444	5·138	4·149	**6·578**	5·92	1·467
Jam, marmalade and other fruit spreads	0·904	0·87	1·208	1·082	1·338	1·264	1·207	**1·204**	1·366	1·219
Honey	0·909	0·975	0·597	0·704	0·918	0·9	0·859	**0·946**	0·905	0·942
Wheat biscuits: plain	−0·368	−0·12	0·245	0	0·158	0·408	0	**−0·353**	−0·026	0·844
Cereal flakes	1·787	3·216	3·653	3·421	2·643	3·182	2·247	**2·385**	2·57	1·904
Flavoured breakfast cereals	0·984	0·758	0·831	0·563	0·798	1·026	0·992	**1·181**	0·946	1·055
Sweet biscuits: plain	0·66	0·707	0·579	0·868	0·849	0·923	0·61	**0·727**	0·918	0·792
Sweet biscuits: not plain	2·375	2·342	2·324	2·03	1·605	2·094	1·997	**1·884**	2·282	1·792
Muesli and cake-type bars	0·927	1·122	1·088	0·986	0·994	0·879	0·671	**0·57**	0·687	0·889
Cakes and puddings	1·266	1·233	1·119	1·131	1·182	1·11	1·09	**1·212**	1·205	1·168
Sweet breads and pancakes	0·331	0·722	0·673	0·767	0·847	0·702	0·662	**0·719**	0·421	0·491
Juice: fruit (no added sugar/100 % fruit juice)	0·879	0·912	0·923	0·889	0·925	0·906	0·821	**0·944**	0·921	0·949
Sauce: tomato or barbecue	1·478	1·133	1·044	1·184	0·981	1·161	1·169	**1·745**	1·472	1·05
Sauce and marinade: sweet style	−1·13	−0·676	1·257	0	0	0·313	0	**0·389**	0	0·281
Sugar: regular table sugar	1·499	2·075	1·491	1·646	1·466	2·368	1·426	**1·783**	1·564	1·451
Chocolate or carob	1·418	1·358	1·623	1·413	1·607	1·382	1·289	**1·131**	1·328	1·814
Ice cream, frozen yogurt and non-dairy alternatives	1·89	1·865	1·601	1·643	1·535	1·363	1·86	**1·655**	1·5	1·802
Yogurt and alternatives, flavoured	1·3	1·402	1·345	1·384	1·363	1·316	1·271	**1·374**	1·355	1·232
Savoury biscuits and snack foods	0	−1·021	0·91	0	0·401	0	0·055	**−1·165**	0·132	0·099
Lollies: regular (not sugarfree)	1·866	1·363	1·762	1·864	1·521	1·635	1·893	**1·85**	1·716	1·385

a Bold values indicate coefficients for best model with minimum root mean square error difference between training (2263·86) and testing (2263·59) data.

Of the twenty-four predictors entered into the regression model, all remained after variable shrinkage. The twenty-two items included discretionary items (i.e. those that do not fit into the five food groups due to their high saturated fat and/or added sugars or salt content^(36)^) such as: icy poles and sorbets; ice creams and frozen yogurts; flavoured breakfast cereals (i.e. chocolate, vanilla or malt-flavoured with added sugar content); snack foods (i.e. potato crisps), sweet biscuits; muesli and cake-type bars; cakes and puddings; sweet breads and pancakes; spreads such as jam and honey; chocolate and lollies; fruit juice (no added sugar); and sugar. Sugar-sweetened beverages (such as soft drinks, cordials and fruit drinks) were not a strong predictor of FSI in the 2-year-old sample. Core (‘healthy’)^(36)^ food items such as custard, nut paste, wheat biscuits and cereal flakes, flavoured yogurts (i.e. fruit flavoured), and plain savoury biscuits also remained as strong predictors of FSI. The secondary analysis showed that for the model including child height and weight (see online supplementary material, Supplemental Table S3), the resultant FFQ items (*n* 21) were similar, however, without sweet style sauces and marinades, and savoury biscuits and snack foods.

#### SMILE-FFQ item reduction at 5 years of age

Figure 2 shows the reduction of the 5-year SMILE-FFQ from ninety-eight items to twenty-six items using data from the training sample (*n* 460). First, the ninety-eight FFQ items were reduced by collapsing twenty-five FFQ items into eight (see online supplementary material, Supplemental Table S1) and removing fifty-one items with near-zero variance (see online supplementary material, Supplemental Table S2). A total of thirty-four items (thirty FFQ items plus child age, sex, weight and height) were entered into the linear regression prediction model. Table 3 shows the model coefficients resulting after variable shrinkage across ten cross-validation runs. The RMSE across the ten runs was 2193·3 ± 37·1 (%EFS, 2·5 ± 0·4 %). The best model was R8, with an RMSE of 2·6 %EFS, meaning that on average a child’s predicted %EFS was overestimated by 2·6 %.

**Table 3:** Model coefficients after variable shrinkage across ten cross-validation runs of the regularised regression-based prediction model, using the training sample (*n* 460) at 5 years^a^

Description	R1	R2	R3	R4	R5	R6	R7	R8	R9	R10
(Intercept)	6·769	8·003	7·013	6·562	6·466	7·048	7·628	**6·894**	7·012	6·78
Child factors										
Child age (at 5 years)	0	0	0	0	0	0	0	**0**	0	0
Child sex	0	0	0	0	0	0	0	**0**	0	0
Child height	0	0	0	0	0	0	0	**0**	0	0
Child weight	0	0	0	0	0	0	0	**0**	0	0
Food frequency items										
Milk: flavoured	1·487	0·439	1·255	1·355	0·865	1·544	1·186	**1·153**	1·197	1·706
Custard: infant and toddler	2·411	0	2·08	0·888	1·241	0·589	2·253	**1·109**	1·932	1·145
Icy poles and sorbet	1·286	1·014	1·253	1·229	1·107	1·08	1·308	**1·067**	1·238	1·149
Nut paste	0	0	0	0	0	0·07	0	**0**	0·389	0
Chocolate, choc nut or carob spreads	1·038	1·13	1·191	0·942	0·958	0·973	0·758	**0·602**	0·754	0·926
Jam, marmalade and other fruit spreads	1·03	0·944	0·993	0·98	1·001	0·89	0·977	**1·065**	0·95	0·976
Honey	0·723	0·729	0·691	0·797	0·773	0·852	0·804	**0·82**	0·793	0·813
Wheat biscuits: plain	0	0	0	0	0	0	0	**0**	0	0
Cereal flakes	0	0	0	0	0	0	0	**0**	0	0
Flavoured breakfast cereals	0·758	1·093	1·011	0·722	1·097	0·781	0·735	**0·728**	0·837	0·934
Sweet biscuits: plain	0·756	0·628	0·885	0·886	1·226	1·062	0·493	**0·826**	0·991	0·781
Sweet biscuits: not plain	1·207	1·251	1·054	1·08	0·917	1·013	1·179	**1·072**	1·095	1·141
Muesli and cake-type bars	0·789	1·081	0·712	0·883	0·64	0·813	1·135	**1·041**	0·627	0·78
Cakes and puddings	0·859	1·034	0·86	0·916	0·877	0·834	0·904	**0·946**	0·848	0·895
Sweet breads and pancakes	0·663	0·8	0·647	0·61	0·554	0·632	0·61	**0·793**	0·567	0·74
Sweet pastry	1·049	0·965	1·112	1·184	1·137	1·425	1·113	**1·273**	1·058	1·212
Soft drink: regular (not sugar-free)	0·668	1·191	0·784	1·24	1·072	0·772	0·708	**0·773**	0·777	0·607
Juice: fruit drink	1·153	0·829	1·262	1·162	1·137	1·101	1·131	**1·165**	1·13	0·733
Juice: fruit (no added sugar/100 % fruit juice)	0·911	0·831	0·872	0·787	0·848	0·872	0·884	**0·771**	0·882	0·738
Milo or Ovaltine in milk drinks	0·037	0·768	0·36	0	0	0	0·456	**0·432**	0·014	0·258
Sauce: tomato or barbecue	0·817	1·128	0·839	1·109	0·878	0·79	0·782	**1·11**	0·868	1·09
Sauce and marinade: sweet style	1·426	0·138	1·475	1·299	0·947	1·092	1·141	**1·349**	0·943	0·262
Mayonnaise: regular	0·671	0	0	0	0	0	0	**0**	0	0
Sugar: regular table sugar	0·872	0·856	0·896	1·544	0·908	0·905	0·855	**0·894**	0·873	0·948
Sugar syrups	1·066	0·476	0·647	0·635	0·964	0·631	0·509	**0·454**	0·831	0·581
Chocolate or carob	0·792	0·792	0·908	0·847	0·76	0·836	0·811	**0·829**	1·123	0·837
Ice cream, frozen yogurt and non-dairy alternatives	1·114	1·36	0·973	1·148	1·315	1·097	1·208	**1·218**	1·094	1·193
Yogurt and alternatives, flavoured	1·162	0·942	1·13	1·141	1·061	1·133	0·982	**1·004**	1·022	1·105
Savoury biscuits and snack foods	3·572	0·148	3·065	2·381	3·118	3·316	2·32	**2·453**	3·686	3·839
Lollies: regular (not sugarfree)	1·162	1·243	1·213	1·11	1·244	1·183	1·333	**1·225**	1·329	1·137

a Bold values indicate coefficients for best model with minimum root mean square error difference between training (2199·41) and testing (2196·15) data.

Of the thirty-four predictors entered into the regression model, twenty-six remained after variable shrinkage. That was, four SMILE-FFQ items (nut paste, wheat biscuits, cereal flakes and mayonnaise) were removed via the regression-based predictive analysis model, in addition to the four child predictors. Items remaining as strong predictors were those as per the 2-year analysis, with additional items including discretionary drinks such as flavoured milk, fruit drink, soft drink and powdered milo/Ovaltine (added to milk drinks) and discretionary foods such as chocolate spreads, sweet-style sauces/marinades, sugar syrups (e.g. maple syrup), and sweet pastries. The sensitivity analysis showed that for the model excluding child height and weight (see online supplementary material, Supplemental Table S4), the resultant FFQ items (*n* 27) were similar; however, they did not include wheat biscuits, cereal flakes and mayonnaise.

### Internal validation of the SMILE-FSS

#### Comparison of the measured and predicted percent energy from free sugars

**Fig. 3:**
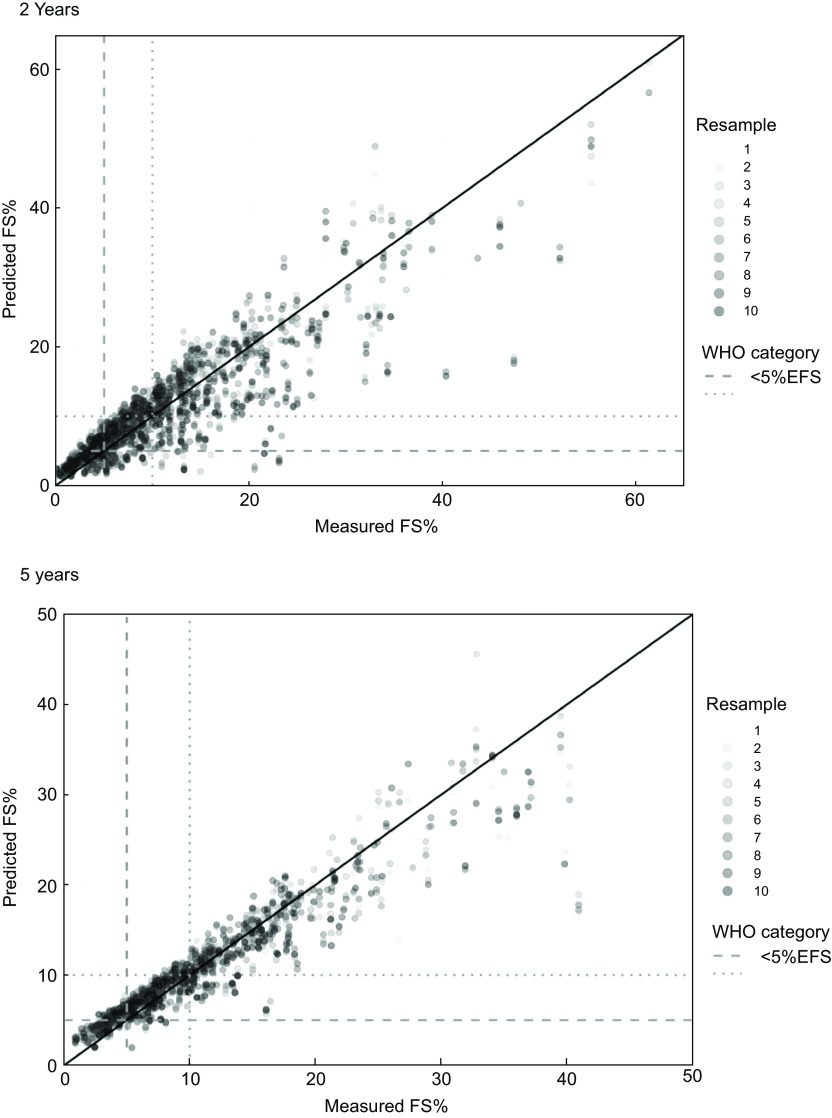
Scatter plot of measured and predicted %EFS with the unity line (representing perfect calibration), using the testing sample (*n* 263 at 2 years; *n* 192 at 5 years). The horizontal and vertical dashed and dotted lines represent the cut-off for < 5 % total energy from free sugars (EFS) and < 10 % total EFS, respectively. EFS, energy from free sugars

Figure 3 shows a scatter plot of the *measured v*. *predicted* %EFS, including a unity line which represents perfect calibration (i.e. where predicted = measured), developed using the testing sample (*n* 263 at 2 years; *n* 193 at 5 years). The figure indicates that at both 2 and 5 years, the models performed worse at predicting larger values of %EFS. The horizontal and vertical dashed and dotted lines represent the cut points for < 5 %EFS and < 10 %EFS, respectively, and indicate that no participant with a %EFS of less than 5 % was predicted to have a %EFS greater than 10 % or vice versa. Supplemental Fig. S3 shows the scatter plot of the measured *v*. predicted FSg, also developed using the testing sample (*n* 263 at 2 years; *n* 193 at 5 years).

#### Comparison to WHO free sugars guideline categories at 2 years of age

Table 4 shows the cross-classification of measured *v*. predicted WHO categorisation of %EFS, in which the column-wise percentages in the diagonal equal the proportion of the participants correctly predicted to be in a given category. At 2 years, a total of 74·9 % were classified into the same category and 25·1 % into an adjacent category (15·6 % over classified and 9·5 % under classified). Correct classification was highest for category 3 (≥ 10 %EFS, 84·5 %), followed by category 1 (< 5 %EFS, 75·9 %) and category 2 (5–< 10 %EFS, 63·2 %). In regard to risk identification (see online supplementary material, Supplemental Table S6), more than four out of five children (*n* 144/166, 87 %) with FSI < 10 %EFS were correctly identified as such, with less than one-quarter (*n* 22/166, 13 %) misclassified as ‘at risk’ (i.e. ≥ 10 %EFS). Conversely, few children (*n* 15/263, 6 %) with FSI ≥ 10 %EFS were not identified as such by the SMILE-FSS (i.e. were predicted to have FSI < 10 %EFS).

**Table 4:** Cross-classification table of measured and predicted WHO free sugars percentage categories^a^, using the testing sample (*n* 263 at 2 years; *n* 192 at 5 years)^b^

Age group	Predicted category	Measured category					
		1		2		3	
2 years	1	**60**	**75·9 %**	10	11·5 %	1	1·0 %
	2	19	24·1 %	**55**	**63·2 %**	14	14·4 %
	3	0	0 %	22	25·3 %	**82**	**84·5 %**
5 years	1	**22**	**56·4 %**	3	3·9 %	0	0 %
	2	17	43·6 %	**68**	**88·3 %**	8	10·5 %
	3	0	0 %	6	7·8 %	**68**	**89·5 %**

a WHO categories: (1) < 5 %EFS, (2) 5–< 10 %EFS and (3) ≥ 10 %EFS.

b Column-wise percentages in the diagonal (in bold) equal the proportion of the participants within each category correctly predicted to be in a given category.

#### Comparison to WHO free sugars guideline categories at 5 years of age

At 5 years, a total of 82·3 % were classified into the same WHO free sugars guideline category and 17·7 % classified into an adjacent category (11·9 % over classified and 5·8 % under classified) (Table 4). Correct classification was highest for category 3 (≥ 10 %EFS, 89·5 %), followed by category 2 (5–< 10 %EFS, 88·3 %) and category 1 (< 5 %EFS, 56·4 %). In regard to risk identification (see online supplementary material, Supplemental Table S6), nearly all children (*n* 110/116, 95 %) with FSI < 10 %EFS were correctly identified as such, with few (*n* 6/116, 5 %) misclassified as ‘at risk’ (i.e. ≥ 10 %EFS). Conversely, few (*n* 8/192, 4 %) children with FSI ≥ 10 %EFS at 5 years were not identified as such by the SMILE-FSS (i.e. were predicted to have FSI < 10 %EFS).

## Discussion

This study employed a regression-based prediction modelling approach to reduce the long-form SMILE-FFQ at 2 and 5 years to a short-form SMILE-FSS. Food items that strongly predicted FSI and were included in the SMILE-FSS (22 and 26 items, respectively) were relatively consistent between age groups, with more discretionary food and beverage items included at 5 years. Internal validation of the SMILE-FSS showed that it performs well in comparison to the long-form SMILE-FFQ at both ages. Most children (75 % and 82 %, respectively) were categorised into the same WHO free sugars category with nearly all children (87 % and 95 %, respectively) correctly identified as having < 10 %EFS in line with the WHO recommendation^(1)^. Overall, the SMILE-FSS was shown to have good internal validity and can be used in future research and practice to estimate total FSI in Australian children aged 2–3 and 5–6 years and compare to the WHO free sugars guidelines to determine those ‘at risk’.

Key predictors of FSI included in the SMILE-FSS were similar at 2 (twenty-two items) and 5 years (twenty-six items) and included discretionary items such as ice cream, sweet biscuits, cakes and puddings, sweet breads and pancakes, sugar-based sauces and spreads, chocolate and lollies. At 5 years (but not 2 years) of age, additional discretionary beverage items such as sugar-sweetened beverages (known as ‘soft drink’ in Australia) and fruit drinks were key predictors of FSI. This is not overly surprising given trends of increasing discretionary beverage intake in the first years of life in Australian children^(37,38)^. For example, a study of Australian children’s beverage intake reported that the proportion of consumers of sweet beverages (including flavoured milk, 100 % juice, fruit drink, cordial and soft drink) increased from approximately one-third (38 %) at 2 years to half at 3·7 (55 %) and 5 (47 %) years^(37)^. A similar trend was observed in a longitudinal study of beverage consumption in Australian children^(38)^, whereby soft drink and cordial consumers increased from 1 % in the first year of life to one-quarter (28 %) at 2 years and nearly half (43 %) at 10 years. Discretionary items such as chocolate spreads and sugar syrups were also additional predictors of FSI at 5 years (but not at 2 years), while core items such as nut paste, wheat (cereal) biscuits (e.g. Weet-Bix, Vita Brits, etc), cereal flakes (e.g. Cornflakes, Wheeties, Sultana Bran, Light n Tasty, etc.) were important in predicting FSI at 2 years (but not at 5 years). Together these findings demonstrate a shift from healthy/core foods as key free sugar contributors in the first few years of life towards greater discretionary food contributors in school-aged children, consistent with population trends^(39)^. Overall, consistency and discrepancies in key free sugar contributors between 2 and 5 years are reflective of changes in patterns of consumption as children move from toddlerhood to childhood.

Importantly, in this study, 100 % fruit juice and flavoured milk ((i.e. chocolate/strawberry milk and milkshakes) were key predictors of FSI at 2 and 5 years, respectively, aligning with the downward trend by age group in fruit juice consumption (78 % 2–3 year olds, 59 % in 4–8 year olds)^(40)^ and the upward trend in flavoured milk consumption^(41)^. However, although the primary goal of the SMILE-FSS is a reduction in FSI for dental health, and thus identification of free sugar containing items is important, 100 % fruit juice and flavoured milk are considered core beverage items within the Australian Dietary Guidelines^(36)^, providing essential nutrients and contributing to children’s fruit and dairy serves^(42)^ This is a particularly important consideration for the 5-year-old SMILE FFS with respect to flavoured milk consumption and dairy serve recommendations given that Australian children older than 4 years of age do not reach recommendations for dairy food intake, consuming ≤ 2 servings/d^(41)^. Thus, end users of the SMILE-FSS should be cautious not to recommend avoidance of these items but rather balance recommendations to reduce FSI with respect to their nutrient contribution.

Validation of the SMILE-FSS showed that the short-form screener has good internal validity. Although a child’s predicted %EFS was overestimated by 4·4 % in 2–3 year olds and by 2·6 % in 5–6 year olds, the SMILE-FSS correctly classified most children (75 % and 82 %, respectively) into the WHO free sugars guidelines categories (< 5, 5–< 10, ≥ 10 %EFS)^(1)^. Further, nearly all children (87 % and 95 %, respectively) were correctly identified as having < 10 %EFS in line with the WHO free sugars recommendation with few children (6 % at 2 years and 4 % at 5 years) with intakes ≥ 10 %EFS (i.e. ‘at risk’) not identified as such by the SMILE-FSS. In contrast, the proportion of children misclassified as ‘at risk’ (i.e. ≥ 10 %EFS) at 2 (13 %) years was higher than that at 5 years (5 %), although similar studies have shown misclassification levels as high as 25 %^(30,31)^.Overall, findings show that the SMILE-FSS performs well in identifying children ‘at risk’ (≥ 10 %EFS) and in need of referral and intervention and can be used in future research to estimate total FSI in Australian children aged 2–3 and 5–6 years.

To our knowledge, no other short dietary-related screening tools exist within the dental context to assess FSI in Australian children. A short-form 8–16 item child oral health-related quality-of-life tool has been developed in a sample of New Zealand 11–14-year-old children, while several short diet-related screening tools (7–28 items) that assess food intake^(28,30–33)^, diet quality^(29)^ and or obesity-related behaviours have been developed and validated in Australian children ranging from 12 months to 16 years of age. These include the twenty-eight-item Children’s Dietary Questionnaire (4–16 years)^(33)^, the nineteen-item Toddler (ages 12–36 months)^(28,30)^ and Preschooler (3–5 years) Dietary Questionnaires^(31)^, and the 7–15-item Early Prevention of Obesity in Childhood Dietary Questionnaire (EPOCH-DQ). None of these tools were designed to capture the leading dietary contributors to dental caries risk, and thus none specifically assess total and/or FSI from major food and beverages sources in young Australian children. The 2- and 5-year-old versions of the SMILE-FSS therefore add to the small yet increasing number of short tools to assess dietary intakes in young Australian children.

The SMILE-FSS is the first short screening tool to assess FSI across the first years of life that is suitable for dental research and practice, showing good internal validity compared with the longer SMILE-FFQ. The SMILE-FSS was developed in a large sample of children (*n* 885 at 2 years and *n* 652 at 5 years) through application of an innovative approach to item selection, a regression-based model utlilising existing data of 2- and 5-year-olds dietary intakes. Other strengths include combining items a priori to ensure the final screener would be fit-for-purpose and the 10-fold cross-validation that was undertaken to determine the best subset of SMILE-FFQ questions that accurately estimate children’s FSI. Child factors (age, sex, height and weight) were added to the model to improve model fit, and sensitivity analyses conducted (with or without height and weight and using both %EFS and FSg to internally validate items in the optimal model) with minimal differences seen between models. Additionally, demographic characteristics of included participants (*n* 885 at 2 years; *n* 652 at 5 years) were compared to those excluded (*n* 158 at 2 years; *n* 64 at 5 years) with no meaningful differences found. However, despite the extensive analyses undertaken in this study to determine internal validity, the external validity of the SMILE-FSS is unknown. This could be determined through completion of the short-form SMILE-FSS and repeat 24-h recalls (the gold standard in dietary assessment)^(43)^ by the same sample to compare FSI of Australian children derived from the two tools. The SMILE-FSS is also limited by the fact that it was developed and internally validated in discrete samples of Australian children aged 2–3 years and 5–6 years, and thus the applicability to children outside of these age ranges is unknown.

Despite these limitations, the SMILE-FSS fills an important gap in the literature, being highly useful in dental research where dietary assessment resources are limited. However, caution should be taken to ensure that core foods that provide nutritional benefits, such as 100 % fruit juice and flavoured milks, are not restricted to ensure young children’s nutritional intake is not compromised. Further, if the SMILE-FSS is to be used in a population substantially different to the one described here, or the tool is modified in any way, further validation (internal or external) would be required to determine its validity within that context. It also has potential, following further work to translate it into a user-friendly online version, to be utilised within the primary healthcare dental setting to screen children’s FSI and support appropriate intervention (i.e. behaviour change messages for oral health regarding reducing and/or limiting sugar consumption) to reduce caries risk^(44)^. Future research, however, is required to determine the feasibility and acceptability (by dental practitioners and caregivers) of this approach^(44)^.

This study used a regression-based prediction modelling approach to develop and internally validate a short twenty-two-item (2 years) and twenty-six-item (5 years) FSS (the SMILE-FSS) using data derived from a longer (99 and 98 items, respectively) FFQ (the SMILE-FFQ). The SMILE-FSS was shown to have good internal validity, accurately estimating FSI and allowing for good comparison to the WHO free sugars guideline categories to determine those ‘at risk’ (i.e. not meeting the < 10 %EFS WHO recommendation). Food items in the SMILE-FSS at both 2 and 5 years are relatively consistent, with more discretionary foods and beverages featuring in the 5-year-SMILE-FSS. In summary, the SMILE-FSS is a novel, age-appropriate, culturally appropriate tool that can be used in research and practice settings to assess FSI of Australian children aged 2–3 and 5–6 years and provide quick assessment against the WHO free sugars guideline. Future research should determine the acceptability and feasibility of implementing the SMILE-FSS within the primary care dental setting.

## Supporting information

Bell et al. supplementary material Bell et al. supplementary material

## Supplementary material

For supplementary material accompanying this paper visit https://doi.org/10.1017/S1368980023002380
