# Supplementary material for: Development and internal validation of the SMILE-FSS: a Free Sugars Screener for Australian children aged 2 and 5 years
Source: Public Health Nutr. 2023 Oct 31;26(12):2691–703. doi: 10.1017/S1368980023002380 (PMC10755434; doi:10.1017/S1368980023002380)
Supplement: Bell et al. supplementary material [file S1368980023002380sup001.docx]

Supplementary files

**Supplementary table S1:** Combining of items from the SMILE-FFQ (*n =* 25 into 8) prior to statistical analysis, based on similarities in food type and free sugars content

| **Original food items, combined prior to statistical analysis** | **Revised food items** |
| --- | --- |
| 1. Yoghurt: plain 2. Yoghurt: plain (reduced fat) 3. Yoghurt alternatives: Plain (all fat) | 1. Yoghurt and alternatives, plain |
| 1. Yoghurt: children's (all fat) 2. Yoghurt: fruit flavours 3. Yoghurt: fruit flavours (reduced fat) 4. Yoghurt: dessert and other flavours 5. Yoghurt: dessert and other flavours (reduced fat) 6. Yoghurt alternatives: flavoured 7. Yoghurt alternatives: flavoured (reduced fat) | 1. Yoghurt and alternatives, flavoured |
| 1. Tinned fruit in syrup, drained 2. Tinned fruit in syrup, undrained 3. Tinned fruit in half syrup, drained 4. Tinned fruit in half syrup, undrained | 1. Tinned fruit in syrup |
| 1. Tinned fruit in juice, drained 2. Tinned fruit in juice, undrained | 1. Tinned fruit in juice |
| 1. Ice-cream and frozen yoghurt 2. Ice-cream alternatives | 1. Ice-cream, frozen yoghurt, and non-dairy alternatives |
| 1. Water: plain, still 2. Water: plain, carbonated | 1. Water, unflavoured, sparkling or still |
| 1. Biscuit, savoury, plain 2. Biscuit, savoury, flavoured 3. Snackfoods: savoury | 1. Savoury biscuits and snack foods |
| 1. Lollies that last a long time 2. Lollies: regular (not sugarfree) | 1. Lollies: regular (not sugarfree) |

**Supplementary table S2:** Summary of variance in consumption of SMILE-FFQ items in complete-case sample (*n =* 885, 2 years; *n =* 652, 5 years), including near-zero variance items removed prior to regularised regression-based prediction modelling

| **FoodID** | **Description** | **2Y** | | | | **5Y** | | | |
| --- | --- | --- | --- | --- | --- | --- | --- | --- | --- |
|  |  | **Frequency Ratio** | **Percent Unique** | **Zero Variance** | **Near-Zero Variance** | **Frequency Ratio** | **Percent Unique** | **Zero Variance** | **Near-Zero Variance** |
| F1_1 | Condensed milk | 291.7 | 0.9 | No | YES | 141.4 | 0.8 | No | YES |
| F1_2 | Evaporated milk | 0.0 | 0.1 | YES | YES | 0.0 | 0.1 | YES | YES |
| F2_1 | Milk: flavoured | 24.4 | 2.4 | No | YES | 9.7 | 2.9 | No | No |
| F2_2 | Milk: plain | 0.0 | 0.1 | YES | YES | 0.0 | 0.1 | YES | YES |
| F2_3 | Milk alternatives: flavoured | 75.0 | 1.9 | No | YES | 43.7 | 2.1 | No | YES |
| F2_4 | Milk alternatives: plain | 35.7 | 3.2 | No | YES | 24.2 | 2.8 | No | YES |
| F2_5 | Infant formula and toddler milk | 23.0 | 2.4 | No | YES | 140.6 | 1.3 | No | YES |
| F3_1 | Cheese: cream or ricotta | 0.0 | 0.1 | YES | YES | 0.0 | 0.1 | YES | YES |
| F3_2 | Cheese: soft, other | 0.0 | 0.1 | YES | YES | 0.0 | 0.1 | YES | YES |
| F3_3 | Cheese: hard | 0.0 | 0.1 | YES | YES | 0.0 | 0.1 | YES | YES |
| F3_4 | Cheese alternatives | 76.5 | 1.7 | No | YES | 67.7 | 1.8 | No | YES |
| F4_5 | Probiotic drink | 73.1 | 1.8 | No | YES | 38.5 | 2.5 | No | YES |
| F5_1 | Sour cream or cream (unsweetened) | 0.0 | 0.1 | YES | YES | 0.0 | 0.1 | YES | YES |
| F5_2 | Custard: infant and toddler | 21.6 | 1.8 | No | YES | 15.2 | 2.1 | No | No |
| F5_3 | Custard: plain or vanilla | 13.3 | 1.9 | No | No | 24.0 | 2.0 | No | YES |
| F5_4 | Desserts: custard, mousse and sweet cream based | 30.6 | 1.7 | No | YES | 29.1 | 2.2 | No | YES |
| F5_5 | Other milk-based desserts | 29.7 | 1.5 | No | YES | 51.2 | 1.4 | No | YES |
| F6_2 | Icy-poles and sorbet | 5.9 | 1.7 | No | No | 3.5 | 2.1 | No | No |
| G1_1 | Fresh fruit | 0.0 | 0.1 | YES | YES | 0.0 | 0.1 | YES | YES |
| G2_1 | Dried fruit: coated | 36.7 | 1.2 | No | YES | 47.7 | 1.4 | No | YES |
| G2_2 | Dried fruit: plain | 0.0 | 0.1 | YES | YES | 0.0 | 0.1 | YES | YES |
| G4_1 | Fruit puree: toddler or infant | 0.0 | 0.1 | YES | YES | - | - | - | - |
| G4_2 | Vegetable puree: toddler or infant | 0.0 | 0.1 | YES | YES | - | - | - | - |
| G4_3 | Fruit puree: regular (commercial) | 48.5 | 1.8 | No | YES | 137.8 | 2.0 | No | YES |
| G4_4 | Fruit puree: home-made (unsweetened) | 0.0 | 0.1 | YES | YES | 0.0 | 0.1 | YES | YES |
| H1_1 | Nuts: coated | 37.9 | 1.5 | No | YES | 31.6 | 1.8 | No | YES |
| H1_2 | Nuts: plain | 0.0 | 0.1 | YES | YES | 0.0 | 0.1 | YES | YES |
| H2_1 | Nut paste | 7.8 | 2.7 | No | No | 7.4 | 3.2 | No | No |
| H2_2 | Chocolate, choc-nut or carob spreads | 21.5 | 2.3 | No | YES | 14.0 | 3.2 | No | No |
| H2_3 | Jam, marmalade and other fruit spreads | 5.8 | 2.5 | No | No | 5.3 | 3.2 | No | No |
| H2_4 | Honey | 9.2 | 2.1 | No | No | 5.4 | 3.5 | Vo | No |
| I1_1 | Porridge or other hot cereal | 0.0 | 0.1 | YES | YES | 0.0 | 0.1 | YES | YES |
| I1_2 | Wheat biscuits: plain | 2.0 | 2.3 | No | No | 3.5 | 2.9 | No | No |
| I1_3 | Puffed, unflavoured cereal | 0.0 | 0.1 | YES | YES | 0.0 | 0.1 | YES | YES |
| I1_4 | Muesli: toasted | 92.4 | 1.7 | No | YES | 47.4 | 1.5 | No | YES |
| I1_5 | Muesli: untoasted | 91.6 | 1.6 | No | YES | 51.5 | 1.8 | No | YES |
| I1_6 | Cereal flakes | 10.5 | 2.0 | No | No | 9.3 | 2.4 | No | No |
| I1_7 | Flavoured breakfast cereals | 10.2 | 1.9 | No | No | 5.5 | 2.9 | No | No |
| J1_1 | Sweet biscuits: plain | 1.1 | 2.7 | No | No | 1.1 | 3.2 | No | No |
| J1_2 | Sweet biscuits: not plain | 9.9 | 2.1 | No | No | 4.8 | 3.4 | No | No |
| J1_3 | Meringue or honeycomb | 174.6 | 0.6 | No | YES | 234.0 | 1.4 | No | YES |
| J2_1 | Snack foods: sweet, cereal based | 40.6 | 1.1 | No | YES | 22.2 | 2.7 | No | YES |
| J2_2 | Muesli and cake-type bars | 11.6 | 2.3 | No | No | 5.8 | 3.2 | No | No |
| J2_3 | Snack foods: fruit-flavoured | 0.0 | 0.1 | YES | YES | 0.0 | 0.1 | YES | YES |
| J3_1 | Cakes and puddings | 1.7 | 1.5 | No | No | 1.1 | 2.1 | No | No |
| J4_1 | Sweet breads and pancakes | 7.0 | 1.8 | No | No | 4.0 | 2.1 | No | No |
| J4_2 | Sweet pastry | 21.7 | 1.1 | No | YES | 11.3 | 1.8 | No | No |
| L1_10 | Soft drink: regular (not sugarfree) | 29.9 | 1.2 | No | YES | 16.9 | 2.1 | No | No |
| L1_11 | Frozen drinks | 77.2 | 1.2 | No | YES | 33.9 | 2.1 | No | YES |
| L1_12 | Tea: leaf | 0.0 | 0.1 | YES | YES | 0.0 | 0.1 | YES | YES |
| L1_13 | Tea: Powder or syrup | 218.8 | 0.7 | No | YES | 175.8 | 1.3 | No | YES |
| L1_14 | Coffee | - | - | - | - | 0.0 | 0.1 | YES | YES |
| L1_3 | Water: lightly flavoured | 83.3 | 1.8 | No | YES | 99.1 | 1.3 | No | YES |
| L1_4 | Juice: infant or toddler | 30.2 | 2.6 | No | YES | 29.0 | 2.2 | No | YES |
| L1_5 | Juice: fruit drink | 23.9 | 2.5 | No | YES | 17.1 | 2.8 | No | No |
| L1_5a | Juice: fruit (no added sugar/100% fruit juice) | 13.0 | 2.7 | No | No | 13.2 | 2.8 | No | No |
| L1_6 | Juice: vegetable | 57.4 | 2.0 | No | YES | 55.4 | 2.2 | No | YES |
| L1_7 | Cordial: sugarfree | 83.9 | 1.6 | No | YES | 136.4 | 2.2 | No | YES |
| L1_8 | Cordial: regular (not sugarfree) | 48.7 | 2.3 | No | YES | 53.0 | 2.8 | No | YES |
| L1_9 | Soft drink: sugarfree | 0.0 | 0.1 | YES | YES | 0.0 | 0.1 | YES | YES |
| L5_1 | Milo or Ovaltine in milk drinks | 32.3 | 2.3 | No | YES | 15.6 | 3.5 | No | No |
| L5_2 | Drinking chocolate in milk drinks | 30.3 | 1.8 | No | YES | 25.1 | 2.8 | No | YES |
| M1_1 | Sauce: tomato or barbecue | 3.2 | 2.8 | No | No | 1.3 | 4.5 | No | No |
| M1_2 | Sauce and marinade: sweet style | 12.6 | 2.0 | No | No | 17.4 | 2.5 | No | No |
| M1_3 | Mayonnaise: low fat | 23.0 | 1.6 | No | YES | 29.0 | 2.4 | No | YES |
| M1_4 | Mayonnaise: regular (not low fat) | 28.6 | 1.6 | No | YES | 17.9 | 2.1 | No | No |
| M1_5 | Chutney or relish | 69.9 | 1.4 | No | YES | 98.0 | 1.3 | No | YES |
| M1_6 | Dessert toppings | 30.7 | 1.5 | No | YES | 20.7 | 2.5 | No | YES |
| N1_1 | Sugar: regular (not sugarfree) | 17.9 | 1.8 | No | No | 14.9 | 2.7 | No | No |
| N1_2 | Sugar syrups | 35.7 | 1.7 | No | YES | 13.9 | 2.5 | No | No |
| N1_3 | Sweeteners | 145.8 | 0.7 | No | YES | 356.5 | 0.4 | No | YES |
| O1_1 | Jelly: sugarfree | 0.0 | 0.1 | YES | YES | 0.0 | 0.1 | YES | YES |
| O1_2 | Jelly: regular (not sugarfree) | 20.7 | 1.9 | No | YES | 20.3 | 2.2 | No | YES |
| O2_1 | Chocolate or carob | 2.0 | 1.8 | No | No | 1.5 | 2.7 | No | No |
| O2_2 | Lollies: sugarfree | 0.0 | 0.1 | YES | YES | 0.0 | 0.1 | YES | YES |
| X1_1 | Ice-cream, frozen yoghurt, and non-dairy alternatives | 2.8 | 4.6 | No | No | 1.2 | 6.1 | No | No |
| X1_2 | Yoghurt and alternatives: plain | 142.3 | 1.6 | No | YES | 139.0 | 1.8 | No | YES |
| X1_3 | Yoghurt and alternatives: flavoured | 4.2 | 32.9 | No | No | 5.8 | 33.9 | No | No |
| X1_4 | Water: unflavoured | 0.0 | 0.1 | YES | YES | 0.0 | 0.1 | YES | YES |
| X1_5 | Savoury biscuits and snack foods | 1.6 | 44.4 | No | No | 1.7 | 54.1 | No | No |
| X1_6 | Lollies: regular (not sugarfree) | 5.8 | 3.2 | No | No | 2.6 | 5.4 | No | No |
| X1_7 | Tinned fruit in syrup | 168.2 | 2.9 | No | YES | 136.6 | 2.9 | No | YES |
| X1_8 | Tinned fruit in juice | 33.6 | 3.1 | No | YES | 56.7 | 3.5 | No | YES |

Frequency Ratio: ratio of frequencies for the most common value over the second most common value; Percent Unique: percentage of unique data points; Zero Variance: i.e., only one distinct value; Near-Zero Variance: <10% unique values or ratio of most common value is >19

**Supplementary table S3:** Characteristics of mother-child dyads in the samples with complete data (included) at 2 (*n =* 885) and 5 (*n =* 652) years versus participants excluded (*n =* 158, 2 years; *n =* 64, 5 years)^a^

|  | **2 Years** | | | |  | | **5 Years** | | | |  | |
| --- | --- | --- | --- | --- | --- | --- | --- | --- | --- | --- | --- | --- |
| **Characteristic** | **Included** | | **Excluded** | | **P-value** | | **Included** | | **Excluded** | | **P-value** | |
| **Child Characteristics** |  | |  | |  | |  | |  | |  | |
| Child's age: Mean (SD) | 2.1 (0.1) | | 2.2 (0.2) | | 0.03* | | 5.3 (0.4) | | 5.2 (0.4) | | 0.004* | |
| Child's sex |  | |  | | 0.69 | |  | |  | | 0.41 | |
| Male | 469 (53.0) | | 87 (55.1) | |  | | 357 (54.8) | | 39 (60.9) | |  | |
| Female | 416 (47.0) | | 71 (44.9) | |  | | 295 (45.2) | | 25 (39.1) | |  | |
| Child_BMI category^b^ |  | |  | | 0.25 | |  | |  | | - | |
| Missing | 118 (13.3) | | 20 (12.7) | |  | | 0 (0.0) | | 64 (100.0) | |  | |
| Healthy weight | 673 (76.1) | | 126 (79.8) | |  | | 448 (68.7) | | 0 (0.0) | |  | |
| Overweight | 71 (8.0) | | 11 (7.0) | |  | | 143 (21.9) | | 0 (0.0) | |  | |
| Obesity | 23 (2.6) | | 1 (0.6) | |  | | 61 (9.4) | | 0 (0.0) | |  | |
| **Maternal and Household Characteristics** | | | | | | | | | |  | |  |
| Mother's age: Mean (SD) | 30.5 (5.0) | | 30.8 (4.7) | | 0.41 | | 30.8 (5.1) | | 31.1 (4.4) | | 0.58 | |
| Mother's BMI category^b^ |  | |  | | 0.01* | |  | |  | | 0.68 | |
| Missing | 59 (6.7) | | 9 (5.7) | |  | | 125 (19.2) | | 18 (28.1) | |  | |
| <25kg/m2 | 476 (53.8) | | 88 (55.7) | |  | | 315 (48.3) | | 27 (42.2) | |  | |
| 25-29.9kg/m2 | 176 (19.9) | | 43 (27.2) | |  | | 122 (18.7) | | 9 (14.1) | |  | |
| ≥30kg/m2 | 174 (19.7) | | 18 (11.4) | |  | | 90 (13.8) | | 10 (15.6) | |  | |
| Mother's country of birth |  | |  | | 0.006* | |  | |  | | 1 | |
| Missing | 8 (0.9) | | 0 (0.0) | |  | | 7 (1.1) | | 1 (1.6) | |  | |
| AU/NZ/UK | 686 (77.5) | | 107 (67.7) | |  | | 480 (73.6) | | 47 (73.4) | |  | |
| Others | 191 (21.6) | | 51 (32.3) | |  | | 165 (25.3) | | 16 (25.0) | |  | |
| Mother's education attainment | |  | | 0.48 | |  | |  | | 0.04* | |  |
| Missing | 0 (0.0) | | 0 (0.0) | |  | | 0 (0.0) | | 0 (0.0) | |  | |
| School/vocational | 379 (42.8) | | 73 (46.2) | |  | | 254 (39.0) | | 16 (25.0) | |  | |
| Some university and above | 506 (57.2) | | 85 (53.8) | |  | | 398 (61.0) | | 48 (75.0) | |  | |
| Annual household income, $AUD | | | | 0.2 | |  | |  | | 0.77 | |  |
| Missing | 7 (0.8) | | 1 (0.6) | |  | | 7 (1.1) | | 0 (0.0) | |  | |
| ≤40K | 97 (11.0) | | 19 (12.0) | |  | | 65 (10.0) | | 7 (10.9) | |  | |
| 40.1-80K | 267 (30.2) | | 52 (32.9) | |  | | 208 (31.9) | | 19 (29.7) | |  | |
| 80.1-120K | 269 (30.4) | | 55 (34.8) | |  | | 216 (33.1) | | 19 (29.7) | |  | |
| >120K | 245 (27.7) | | 31 (19.6) | |  | | 156 (23.9) | | 19 (29.7) | |  | |
| Index of Relative Socio-Economic Advantage and Disadvantage | | | | 0.11 | |  | |  | | 0.53 | |  |
| Missing | 7 (0.8) | | 0 (0.0) | |  | | 5 (0.8) | | 3 (4.7) | |  | |
| Deciles 1-2 | 203 (22.9) | | 52 (32.9) | |  | | 146 (22.4) | | 11 (17.2) | |  | |
| Deciles 3-4 | 231 (26.1) | | 38 (24.1) | |  | | 161 (24.7) | | 12 (18.8) | |  | |
| Deciles 5-6 | 151 (17.1) | | 26 (16.5) | |  | | 120 (18.4) | | 14 (21.9) | |  | |
| Deciles 7-8 | 220 (24.9) | | 32 (20.3) | |  | | 166 (25.5) | | 16 (25.0) | |  | |
| Deciles 9-10 | 73 (8.2) | | 10 (6.3) | |  | | 54 (8.3) | | 8 (12.5) | |  | |
| Number of children at home | |  | | 0.62 | |  | |  | | 0.88 | |  |
| Missing | 25 (2.8) | | 5 (3.2) | |  | | 19 (2.9) | | 4 (6.2) | |  | |
| 1 | 413 (46.7) | | 67 (42.4) | |  | | 317 (48.6) | | 32 (50.0) | |  | |
| 2 | 310 (35.0) | | 59 (37.3) | |  | | 232 (35.6) | | 21 (32.8) | |  | |
| ≥3 | 137 (15.5) | | 27 (17.1) | |  | | 84 (12.9) | | 7 (10.9) | |  | |
| Two parent household |  | |  | | 1 | |  | |  | | 0.54 | |
| Missing | 4 (0.5) | | 0 (0.0) | |  | | 5 (0.8) | | 1 (1.6) | |  | |
| Single parent household | 47 (5.3) | | 8 (5.1) | |  | | 31 (4.8) | | 4 (6.2) | |  | |
| Two-parent household | 834 (94.2) | | 150 (94.9) | |  | | 616 (94.5) | | 59 (92.2) | |  | |

Abbreviations: BMI, Body Mass Index; NZ, New Zealand; UK, United Kingdom;

^a^Excluded participants refers to those with complete and plausible dietary data but incomplete sociodemographic data. Data presented as N (%) unless otherwise stated

^b^ Weight status categories equivalent to body mass index (BMI) (kg/m2); Healthy weight <25kg/m2, Overweight 25–29.9 kg/m2, Obesity ≥30 kg/m2.

*denotes statistical significance, p<0.05

**Supplementary table S4:** Model coefficients including child height and weight after variable shrinkage across 10 cross-validation runs of the regularised regression-based prediction model, using the training sample (*n* = 622) at **2 years^a^**

| **Description** | **R1** | **R2** | **R3** | **R4** | **R5** | **R6** | **R7** | **R8** | **R9** | **R10** |
| --- | --- | --- | --- | --- | --- | --- | --- | --- | --- | --- |
| (Intercept) | -2.789 | -3.7 | -7.271 | -9.401 | **-13.239** | 1.407 | -2.902 | -0.277 | 1.523 | 1.319 |
| *Child factors* |  |  |  |  |  |  |  |  |  |  |
| Child age (at 2 years) | 5.777 | 7.123 | 5.505 | 7.621 | **7.707** | 2.343 | 3.868 | 2.217 | 2.228 | 2.94 |
| Child sex | -1.258 | -0.817 | -0.788 | -0.888 | **0** | 0 | 0 | -0.001 | -0.002 | -0.565 |
| Child height | -0.057 | -0.084 | 0 | 0 | **0** | 0 | -0.009 | 0 | 0 | 0 |
| Child weight | 0 | 0.111 | 0.075 | 0 | **0.15** | 0 | 0 | -0.031 | 0 | -0.064 |
| *Food frequency items* |  |  |  |  |  |  |  |  |  |  |
| Custard: plain or vanilla | 1.446 | 1.242 | 1.22 | 0.776 | **0.854** | 0.972 | 0.957 | 0.775 | 0.819 | 0.734 |
| Icy-poles and sorbet | 1.798 | 1.545 | 1.774 | 1.505 | **2.031** | 1.58 | 1.725 | 1.568 | 1.68 | 0.929 |
| Nut paste | 6.431 | 0.187 | 5.766 | 0 | **6.273** | 6.141 | 1.915 | 4.721 | 4.745 | 0 |
| Jam, marmalade and other fruit spreads | 1.07 | 0.688 | 0.793 | 0.83 | **1.117** | 0.979 | 1.346 | 0.585 | 0.907 | 1.012 |
| Honey | 0.656 | 0.876 | 0.879 | 0.784 | **0.826** | 0.29 | 0.918 | 0.859 | 0.407 | 0.597 |
| Wheat biscuits: plain | 0.057 | 0.699 | 0 | 0 | **1.272** | 0.428 | 1.262 | 2.124 | 0.439 | 0 |
| Cereal flakes | 2.603 | 2.411 | 3.31 | 1.799 | **1.873** | 2.619 | 2.688 | 2.39 | 2.743 | 2.391 |
| Flavoured breakfast cereals | 1.005 | 0.912 | 0.465 | 1.048 | **0.915** | 0.74 | 0.7 | 0.898 | 0.598 | 0.973 |
| Sweet biscuits: plain | 0.602 | 0.622 | 0.48 | 0.559 | **0.63** | 0.76 | 0.658 | 0.619 | 0.323 | 0.689 |
| Sweet biscuits: not plain | 2.145 | 2.009 | 2.344 | 2.664 | **2.182** | 2.354 | 2.105 | 1.775 | 2.508 | 2.259 |
| Muesli and cake-type bars | 1.071 | 0.874 | 0.996 | 0.922 | **0.987** | 0.891 | 0.819 | 0.938 | 1.04 | 0.841 |
| Cakes and puddings | 1.197 | 1.236 | 1.134 | 1.079 | **1.186** | 1.036 | 1.104 | 1.112 | 1.091 | 1.058 |
| Sweet breads and pancakes | 0.542 | 0.792 | 0.704 | 0.687 | **0.702** | 0.599 | 0.869 | 1.058 | 0.819 | 1.083 |
| Juice: fruit (no added sugar/100% fruit juice) | 0.92 | 0.87 | 0.819 | 0.712 | **0.809** | 0.756 | 0.893 | 0.777 | 0.757 | 0.72 |
| Sauce: tomato or barbecue | 1.723 | 0.876 | 1.443 | 1.163 | **0.966** | 0.578 | 1.329 | 0.86 | 1.181 | 0.925 |
| Sauce and marinade: sweet style | 0 | 0 | -0.274 | 0 | **0** | 0 | -0.005 | 0.014 | 0 | 0 |
| Sugar: regular table sugar | 2.277 | 2.898 | 2.055 | 2.15 | **2.173** | 3.117 | 3.089 | 3.31 | 2.363 | 1.923 |
| Jelly: regular (not sugarfree) | 2.684 | 1.105 | 0.94 | 1.893 | **1.55** | 1.101 | 1.271 | 2.257 | 1.013 | 2.074 |
| Chocolate or carob | 1.19 | 1.355 | 1.41 | 1.189 | **1.169** | 1.424 | 1.223 | 1.478 | 1.138 | 1.45 |
| Ice-cream, frozen yoghurt, and non-dairy alternatives | 1.939 | 1.506 | 1.192 | 1.475 | **1.689** | 1.087 | 0.81 | 1.369 | 1.731 | 1.624 |
| Yoghurt and alternatives, flavoured | 1.159 | 1.236 | 1.233 | 1.168 | **1.185** | 1.117 | 1.251 | 1.289 | 1.166 | 1.094 |
| Savoury biscuits and snack foods | -0.088 | 0.238 | 0 | 0 | **0** | 0.038 | 0.446 | 0.483 | 0.186 | 0.627 |
| Lollies: regular (not sugarfree) | 1.687 | 1.441 | 1.731 | 1.847 | **1.4** | 1.643 | 1.974 | 1.71 | 1.752 | 1.244 |

^a^Bold values indicate coefficients for best model with minimum Root Mean Square Error difference between training (2245.92) and testing (2238.71) data

**Supplementary table S5:** Model coefficients excluding child height and weight after variable shrinkage across 10 cross-validation runs of the regularised regression-based prediction model, using the training sample (*n* = 460) at 5 years^a^

| **Description** | **R1** | **R2** | **R3** | **R4** | **R5** | **R6** | **R7** | **R8** | **R9** | **R10** |
| --- | --- | --- | --- | --- | --- | --- | --- | --- | --- | --- |
| (Intercept) | **7.271** | 7.139 | 7.203 | 6.639 | 6.846 | 7.792 | 6.84 | 6.786 | 6.776 | 6.906 |
| *Child factors* |  |  |  |  |  |  |  |  |  |  |
| Child age (at 5 years) | **0** | 0 | 0 | 0 | 0 | 0 | 0 | 0 | 0 | 0 |
| Child sex | **0** | 0 | 0 | 0 | 0 | 0 | 0 | 0 | 0 | 0 |
| *Food frequency items* |  |  |  |  |  |  |  |  |  |  |
| Milk: flavoured | **0.982** | 0.989 | 1.444 | 1.694 | 1.41 | 0.875 | 1.398 | 1.383 | 1.236 | 0.772 |
| Custard: infant and toddler | **0.757** | 1.02 | 0 | 1.142 | 0.747 | 0 | 0.89 | 1.046 | 0.727 | 0.754 |
| Icy-poles and sorbet | **1.003** | 1.075 | 1.049 | 1.048 | 1.017 | 1.157 | 0.78 | 0.975 | 1.163 | 0.982 |
| Nut paste | **0.372** | 0.009 | 0.277 | 0 | 0.141 | 0.004 | 0.402 | 0 | 0 | 0 |
| Chocolate, choc-nut or carob spreads | **0.799** | 0.496 | 0.842 | 0.776 | 0.786 | 1.099 | 0.976 | 0.914 | 0.968 | 0.978 |
| Jam, marmalade and other fruit spreads | **0.915** | 1.045 | 1.157 | 1.098 | 1.014 | 0.914 | 1 | 0.813 | 0.927 | 0.927 |
| Honey | **0.807** | 0.74 | 0.694 | 0.76 | 0.785 | 0.761 | 0.814 | 0.79 | 0.78 | 0.776 |
| Wheat biscuits: plain | **0** | 0 | 0 | 0 | 0 | 0 | 0 | 0 | 0 | 0 |
| Cereal flakes | **0** | 0 | 0 | 0 | 0 | 0 | 0 | 0 | 0 | 0 |
| Flavoured breakfast cereals | **0.772** | 0.943 | 0.843 | 0.994 | 0.922 | 0.721 | 0.913 | 0.813 | 0.728 | 0.836 |
| Sweet biscuits: plain | **0.86** | 1.142 | 1.041 | 1.236 | 0.939 | 0.886 | 1.039 | 1.122 | 0.935 | 0.946 |
| Sweet biscuits: not plain | **1.033** | 1.067 | 0.989 | 0.96 | 1.172 | 1.138 | 1.157 | 1.119 | 0.944 | 0.97 |
| Muesli and cake-type bars | **0.869** | 0.761 | 0.537 | 0.786 | 0.818 | 0.847 | 0.848 | 0.662 | 0.705 | 1.038 |
| Cakes and puddings | **0.803** | 0.869 | 0.987 | 0.925 | 0.839 | 0.93 | 0.925 | 0.914 | 0.942 | 1.001 |
| Sweet breads and pancakes | **0.656** | 0.848 | 0.4 | 0.514 | 0.628 | 0.641 | 0.561 | 0.632 | 0.713 | 0.5 |
| Sweet pastry | **1.134** | 0.937 | 1.254 | 0.851 | 1.16 | 0.729 | 0.991 | 1.079 | 1.924 | 1.663 |
| Soft drink: regular (not sugarfree) | **1.113** | 0.841 | 0.698 | 1.179 | 0.701 | 0.931 | 1.084 | 0.633 | 0.804 | 0.62 |
| Juice: fruit drink | **1.11** | 1.097 | 1.128 | 1.241 | 1.324 | 1.109 | 1.105 | 1.125 | 1.165 | 0.933 |
| Juice: fruit (no added sugar/100% fruit juice) | **0.842** | 0.873 | 0.835 | 0.882 | 0.872 | 0.836 | 0.754 | 0.855 | 0.827 | 0.802 |
| Milo or Ovaltine in milk drinks | **0.413** | 0 | 0.367 | 0.212 | 0.139 | 0.579 | 0.094 | 0 | 0.35 | 0.551 |
| Sauce: tomato or barbecue | **1.233** | 0.878 | 0.842 | 0.889 | 0.825 | 1.077 | 1.141 | 0.842 | 0.945 | 1.07 |
| Sauce and marinade: sweet style | **1.073** | 0.62 | 1.92 | 1.131 | 1.164 | 0.925 | 1.424 | 1.836 | 1.506 | 1.994 |
| Mayonnaise: regular | **0** | 0 | 0 | 0 | 0 | 0 | 0 | 0 | 0.016 | 0 |
| Sugar: regular table sugar | **1.293** | 0.874 | 0.909 | 0.848 | 0.916 | 0.808 | 0.904 | 1.598 | 1.377 | 0.893 |
| Sugar syrups | **0.342** | 0.588 | 0.9 | 0.617 | 0.577 | 0.35 | 0.583 | 0.272 | 0.417 | 0.553 |
| Chocolate or carob | **0.853** | 0.989 | 0.821 | 0.8 | 0.792 | 0.908 | 0.778 | 0.765 | 0.803 | 0.817 |
| Ice-cream, frozen yoghurt, and non-dairy alternatives | **1.281** | 1.124 | 1.232 | 1.042 | 1.221 | 1.247 | 1.085 | 1.203 | 1.174 | 1.444 |
| Yoghurt and alternatives, flavoured | **1.088** | 1.15 | 1.048 | 1.132 | 1.102 | 0.999 | 1.206 | 1.085 | 1.012 | 0.999 |
| Savoury biscuits and snackfoods | **2.428** | 2.134 | 2.782 | 2.263 | 3.049 | 0.704 | 2.655 | 3.581 | 3.098 | 2.468 |
| Lollies: regular (not sugarfree) | **1.169** | 1.041 | 1.324 | 1.184 | 1.311 | 1.294 | 1.224 | 1.255 | 1.209 | 1.24 |

^a^Bold values indicate coefficients for best model with minimum Root Mean Square Error difference between training (2192.71) and testing (2203.75) data

**2 years**

**
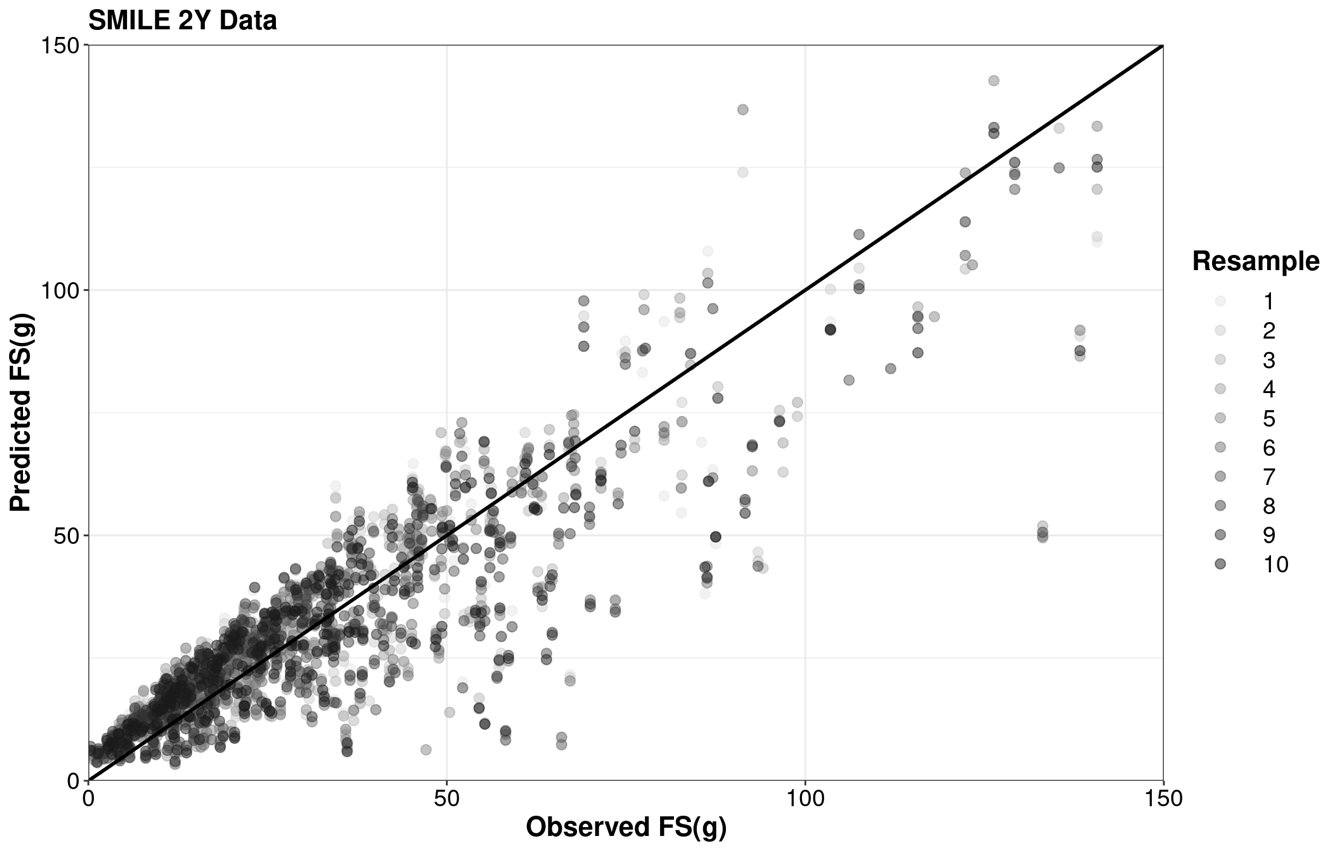
**

**Measured FSg**

<10%EFS

**5 years**

**
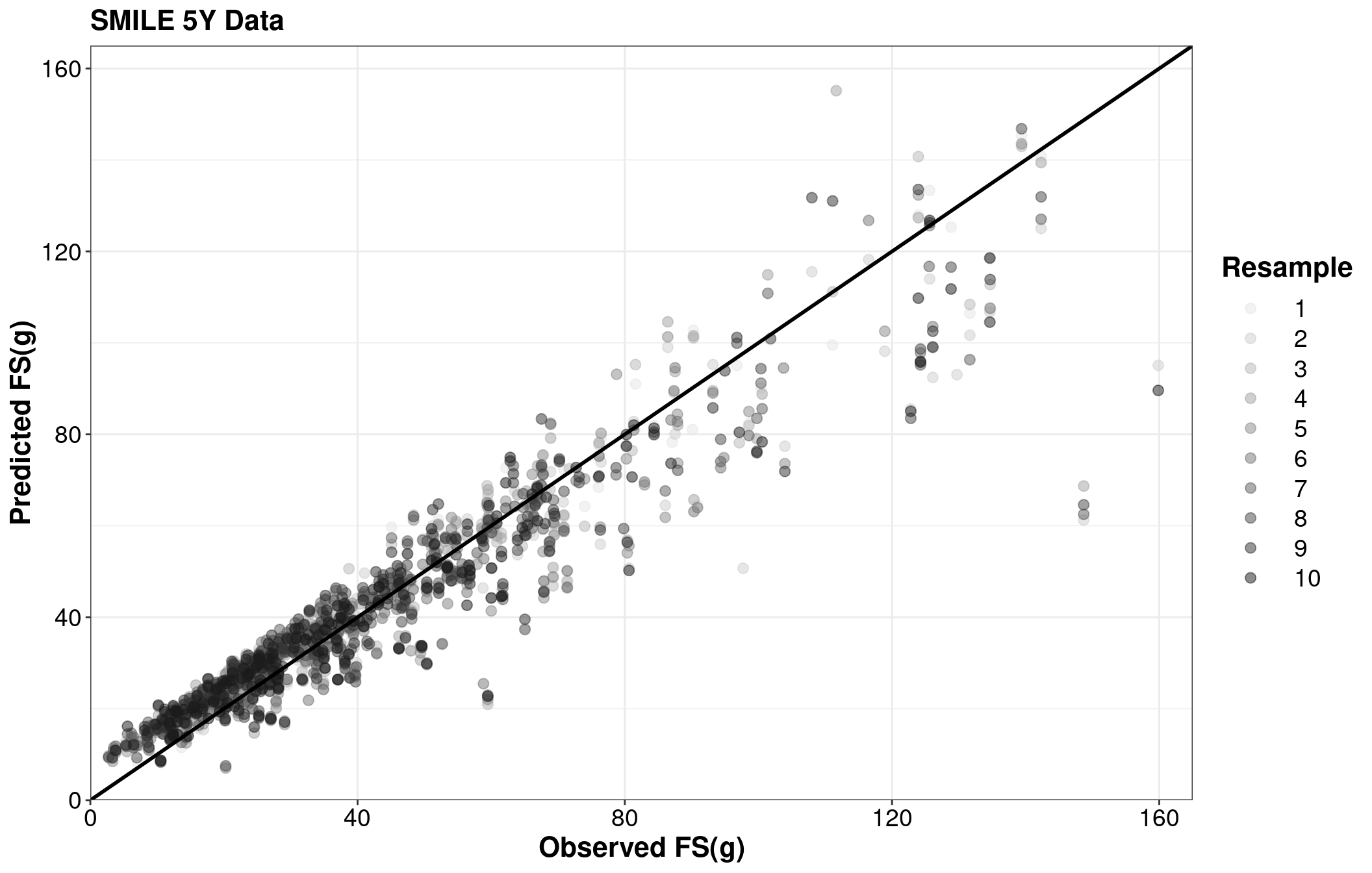
**

**Measured FSg**

<10%EFS

**Supplementary Figure S3:** Scatter plot of measured and predicted Free Sugars intake in grams (FSg) with the unity line (representing perfect calibration), using the testing sample (*n =* 263 at 2 years; *n =* 192 at 5 years).

**Supplementary Table S6:** Cross-classification table of measured and predicted WHO free sugars percentage categories^a^, using the testing sample (*n =* 263 at 2 years; *n =* 192 at 5 years) ^b o^

|  |  |  | | |  |
| --- | --- | --- | --- | --- | --- |
| **Age group** | **Predicted category** | **Measured category** | | |  |
|  |  | 1 | 2 | 3 |  |
| 2 years | 1 | **60 (22.8%)** | 10 (3.8%) | 1 (0.4%) |  |
|  | 2 | 19 (7.2%) | **55 (20.9%)** | 14 (5.3%) |  |
|  | 3 | 0 (0%) | 22 (8.4%) | **82 (31.2%)** |  |
|  |  | **30.0%** | **33.0%** | **36.9%** | **100%** |
| 5 years | 1 | **22 (11.4%)** | 3 (1.6%) | 0 (0%) |  |
|  | 2 | 17 (8.8%) | **68 (35.4%)** | 8 (4.2%) |  |
|  | 3 | 0 (0%) | 6 (3.1%) | **68 (35.4%)** |  |
|  |  | **20.3%** | **40.1%** | **39.6%** | **100%** |

^a^WHO categories: (1) <5%EFS, (2) 5-<10%EFS, (3) ≥10%EFS

^b^column-wise percentages in the diagonal equal the proportion of the participants in the **whole sample** correctly predicted to be in a given category
